# *SERPINA2* Is a Novel Gene with a Divergent Function from *SERPINA1*

**DOI:** 10.1371/journal.pone.0066889

**Published:** 2013-06-24

**Authors:** Patrícia Isabel Marques, Zélia Ferreira, Manuella Martins, Joana Figueiredo, Diana Isabel Silva, Patrícia Castro, Ramiro Morales-Hojas, Joana Simões-Correia, Susana Seixas

**Affiliations:** 1 Institute of Molecular Pathology and Immunology of the University of Porto, Porto, Portugal; 2 Institute of Biomedical Sciences Abel Salazar, University of Porto, Porto, Portugal; 3 Department of Biology, Faculty of Sciences, University of Porto, Porto, Portugal; 4 Medical Faculty, University of Porto, Porto, Portugal; 5 Molecular Evolution, Institute of Molecular and Cell Biology, University of Porto, Porto, Portugal; 6 Institute of Biomedical Research on Light and Image, Faculty of Medicine, University of Coimbra, Coimbra, Portugal; National Institute for Medical Research, Medical Research Council, United Kingdom

## Abstract

Serine protease inhibitors (SERPINs) are a superfamily of highly conserved proteins that play a key role in controlling the activity of proteases in diverse biological processes. The *SERPIN* cluster located at the 14q32.1 region includes the gene coding for SERPINA1, and a highly homologous sequence, *SERPINA2*, which was originally thought to be a pseudogene. We have previously shown that *SERPINA2* is expressed in different tissues, namely leukocytes and testes, suggesting that it is a functional SERPIN. To investigate the function of *SERPINA2,* we used HeLa cells stably transduced with the different variants of SERPINA2 and SERPINA1 (M1, S and Z) and leukocytes as the *in vivo* model. We identified SERPINA2 as a 52 kDa intracellular glycoprotein, which is localized at the endoplasmic reticulum (ER), independently of the variant analyzed. SERPINA2 is not significantly regulated by proteasome, proposing that ER localization is not due to misfolding. Specific features of SERPINA2 include the absence of insoluble aggregates and the insignificant response to cell stress, suggesting that it is a non-polymerogenic protein with divergent activity of SERPINA1. Using phylogenetic analysis, we propose an origin of *SERPINA2* in the crown of primates, and we unveiled the overall conservation of *SERPINA2* and *A1*. Nonetheless, few SERPINA2 residues seem to have evolved faster, contributing to the emergence of a new advantageous function, possibly as a chymotrypsin-like SERPIN. Herein, we present evidences that *SERPINA2* is an active gene, coding for an ER-resident protein, which may act as substrate or adjuvant of ER-chaperones.

## Introduction

The superfamily of serine protease inhibitors (SERPINs) comprises a large number of proteins widely distributed among animals, plants, viruses, and bacteria, characterized by a conserved highly-order tertiary structure. In general, SERPINs act as inhibitors of serine or cysteine proteases in diverse biological process such as coagulation, fibrinolysis, angiogenesis, inflammation and apoptosis. However, a small fraction of SERPINs exert other roles outside of proteolysis regulation for example as molecular chaperones, hormone transporters or storage proteins [1]–[4].

Mechanistically, the inhibitory properties of SERPINs are correlated with their ability to undergo a striking conformational transition (“stressed” to “relaxed” transition). SERPINs present a pseudosubstrate in an exposed reactive centre loop (RCL) able to entrap proteases. Once cleaved by the target protease, the RCL moves to the opposite pole of the molecule, through a β-sheet arrangement – the shutter; distorting the protease structure and thereby causing its irreversible loss of catalytic activity [2], [5]. Under physiological conditions, the plasticity of SERPINs allows these proteins to adopt divergent conformations and assembly states. Conversely, it also renders SERPINs sensitive to single mutations of which there are many altering protein folding, biosynthesis and functional activity [5], [6]. In the human α1-antitrypsin (SERPINA1), the major protease inhibitor in the serum, this molecular vulnerability is well illustrated by the Z allele. This variant results from the amino acid replacement E342K, and affects the RCL leading to a decrease of inhibitory activity and to the polymerization and accumulation of SERPINA1 (∼80%) in the endoplasmic reticulum (ER) [1], [2]. The deficiency of α1-antitrypsin is mainly associated to the ZZ genotype and it affects 1 in 2,000 to 1 in 7,000 individuals of European descend [7], [8]. The major clinical manifestations of the disease are the early pulmonary emphysema, due to the unopposed action of the neutrophil elastase in the lower respiratory tract and the hepatic disease caused by the cytotoxic effect of protein aggregation in hepatocytes [5]–[8].

Apart from the Z allele, other common variants of SERPINA1 are described and include the M1, M2, and M3 alleles, linked to normal circulating protein levels; and the S allele, which results from the amino acid replacement E264V at the shutter region of the molecule [1], [9]. To a lower extent, the S allele also leads to the accumulation of the misfolded protein in the ER (∼50%), however its association to disease is mainly restricted to the lung pathology, and mostly in smokers with SZ genotype [8], [10].

The gene encoding SERPINA1 is located on chromosome 14q32.1 in a gene cluster comprising 10 additional members of the *SERPIN* superfamily (*A2*, *A3*, *A4*, *A5*, *A6*, *A9*, *A10*, *A11*, *A12* and *A13*) [11], [12]. Most genes present a common organization, with one untranslated exon and four coding exons, typical of α1-antytrypsin-like SERPINs (clade A), suggesting that they evolved from a common ancestral gene through a series of duplication events [13], [14]. With the exception of *SERPINA2,* which has a high sequence similarity to *SERPINA1* (∼80% sequence identity) all other genes are likely to represent ancient events of SERPIN diversification [13]–[15].

*SERPINA2* was originally thought to be a pseudogene because no promoter region or liver expression was detected, and a significant level of sequence degeneration was observed, which included a disrupted starting codon (ATG to ATA) and a 2 kb deletion encompassing exon IV and part of exon V [15], [16]. Currently, several lines of evidence indicate that *SERPINA2* has an active isoform and is differently expressed from its closest homologue, *SERPINA1: SERPINA2* is mostly expressed in the testes and leukocytes, having a residual expression in the liver; whereas *SERPINA1* is highly expressed in the liver, has lower expression rates in leukocytes and no measurable expression in the testes [17]. In addition, translation of the full transcript of *SERPINA2* is predicted to encode a regular SERPIN with a distinct inhibitory activity from SERPINA1– reactive site (P1-P1’) composed by tryptophan and serine instead of methionine and serine [15], [17]. On the other hand, the inactive form of *SERPINA2* has two disrupting mutations (ATA and 2 kb deletion) in strong linkage disequilibrium, and it has no recognizable transcript. In African populations, this inactive isoform was found to be associated with too little genetic variation for its given frequency (∼ 58%), as if it was favoured by natural selection acting on a beneficial variant. Furthermore, the loss of SERPINA2 was proposed as an ongoing adaptive process possibly associated to an advantageous role in fertility or in host–pathogen interactions [17].

Accordingly, it is eminent to address whether *SERPINA2* is a true gene, determining to which extent it has diverged from *SERPINA1*. To achieve this, we combined a series of molecular and cellular assays using HeLa cells stably expressing different variants of SERPINA2 and A1 and in leukocytes, with a set of phylogenetic analysis performed using multiple coding sequences of *SERPINA2* and *A1* homologues.

## Results

### Expression of SERPINA2 and SERPINA1 in HeLa Cell Line

To characterize the properties of SERPINA2 we generated HeLa cell lines stably expressing recombinant forms of SERPINA2 and A1 linked to a V5-tag (∼3 kDa). A total of seven cell lines were obtained, these included the empty vector (mock), three cells lines expressing different SERPINA2 variants detected in a testes cDNA library: V1 (P308–K320); V2 (L308–E320); and V3 (P308–E320); and three cells lines expressing the SERPINA1 variants: M1 (V213-E264-E342), S (V213-V264-E342) and Z (A213-E264-K342) (Figure S1). No traces of *SERPINA1* or *A2* expression were detected in primary HeLa cell lines (Figure S2).

**Figure 1:**
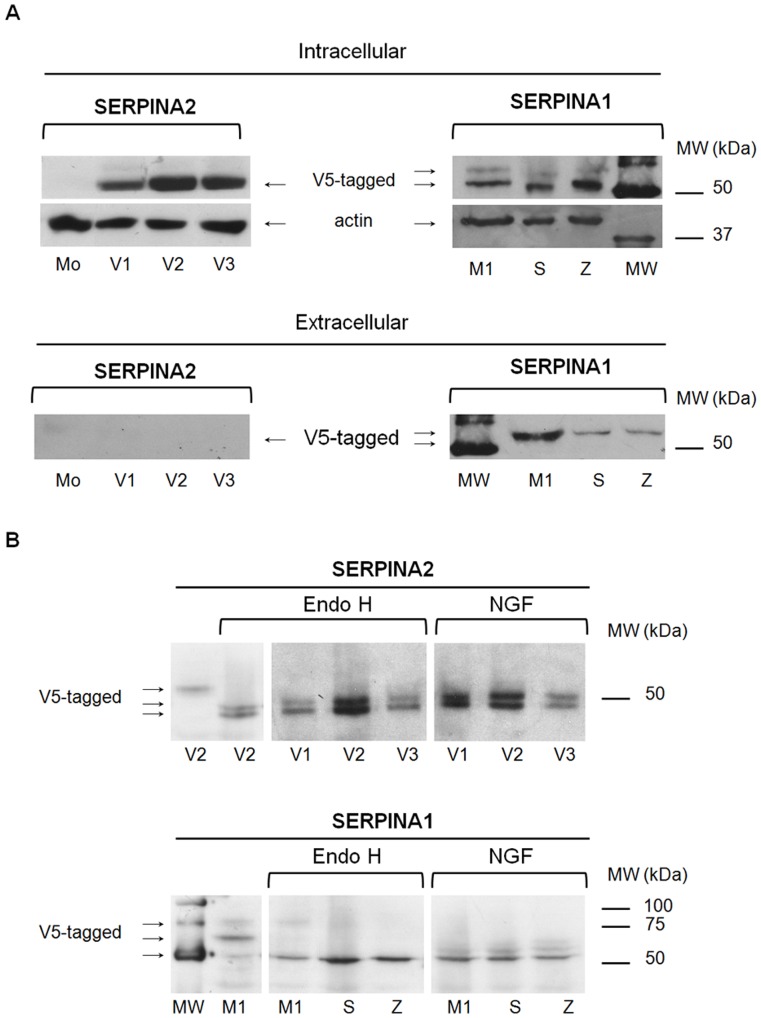
Expression of SERPINA2 and SERPINA1 recombinants. **A** - HeLa cells were stably transduced with the empty vector (Mo) and with *SERPINA2* (V1, V2 and V3) and *SERPINA1* (M1, S and Z) vectors. Intracellular lysates and extracellular media were separated by 10% SDS-PAGE. Proteins were detected with anti-V5 and anti-actin antibodies. **B** - Intracellular lysates were incubated overnight with Endo H and NGF enzymes. Protein were separated by 12% SDS-PAGE and detected with anti-V5 antibody. MW - Molecular weight ladder.

The expression of SERPINA2 and A1 was evaluated at intracellular and extracellular level by Western Blot, using a monoclonal antibody against V5-tag (Figure 1A). In contrast to SERPINA1, which could be detected at both levels, SERPINA2 was detected exclusively in the intracellular fraction (cell lysates). For SERPINA1 two bands corresponding to the partially glycosylated (∼55 kDa) and to the fully glycosylated (∼58 kDa) proteins were observed at the intracellular level, while for SERPINA2 a single band was identified (∼55 kDa). At the extracellular level, only the fully glycosylated (∼58 kDa) band corresponding to the mature protein was detected for SERPINA1, with expected differences in secretion levels of the different variants analyzed (Figure 1A). After the enzymatic treatment with Endoglycosydase H (Endo H) and N-glycosydase F (NGF), the partially glycosylated immature form of SERPINA1 was reduced to approximately 49 kDa, while the fully glycosylated forms were resistant to Endo H and reduced only by NGF. The single form of SERPINA2 was reduced by both enzymes into two bands of 47 kDa and 49 kDa (Figure 1B), suggesting that all the variants of SERPINA2 are partially glycosylated.

### Subcellular Localization of SERPINA2 and SERPINA1

**Figure 2:**
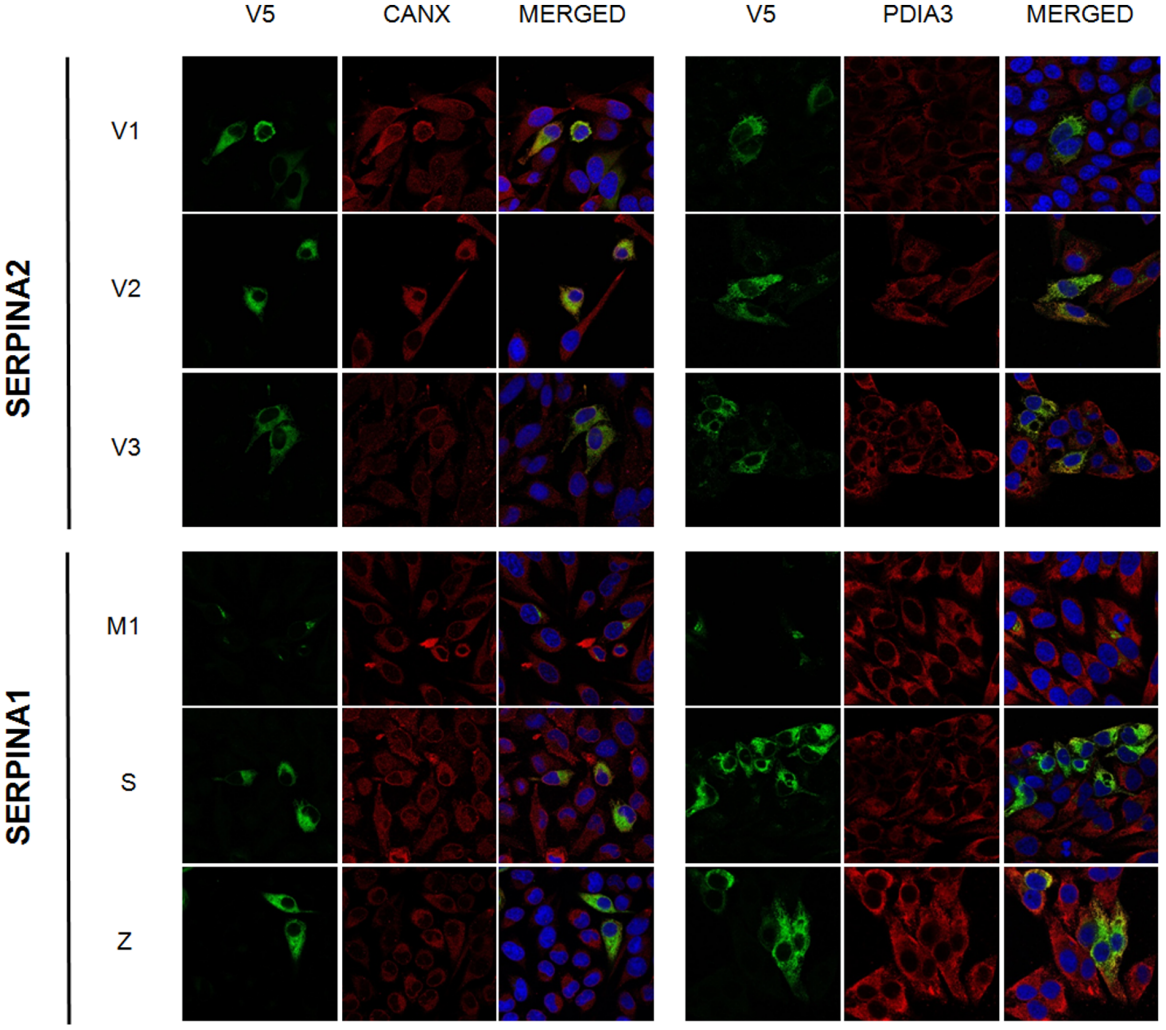
Subcellular localization of SERPINA2 and SERPINA1. HeLa cells were stably transduced with *SERPINA2* (V1, V2 and V3) and *SERPINA1* (M1, S and Z) vectors. SERPINA2 and A1 were stained with anti-V5 and Alexa Fluor 488 (green) antibodies. ER chaperones (CANX or PDIA3) were detected with anti-CANX or anti-PDIA3 and Alexa Fluor 594 (red) antibodies. Nuclei were stained with DAPI. Magnification 400× (confocal microscopy).

To determine the intracellular localization of SERPINA2, the stably transduced Hela cells were subjected to different immunofluorescence assays (Figure 2; Figure S3). The results of the staining with the antibody against V5-tag show that all SERPINA2 variants have a cellular pattern similar to those of S and Z variants (pattern also observed in CHO cells; Figure S4). Except for the M1, in which the staining is concentrated in the perinuclear region and associated to the Golgi apparatus (Figure S5), all other variants present a reticular-like staining (Figure 2, Figure S3).

To address if SERPINA2 was accumulating in the ER, we carried out double-labeling assays against the V5-tag and two ER markers, calnexin (CANX) or Protein Disulfide-Isomerase A3 (PDIA3). The positive overlap with the ER markers in cells expressing SERPINA2 was similar to those of S and Z variants, confirming the localization of SERPINA2 in the ER (Figure 2, Figure S3).

To confirm the subcellular localization of SERPINA2 in the ER we isolated proteins from cytoplasmatic and membranous fractions, the last containing the plasma membrane, ER and Golgi compartments. The segregation of SERPINA2 in the membrane fraction together with a known ER protein provides further support for its localization in ER structures (Figure S6). SERPINA2 variants and S and Z variants of SERPINA1 were found as a single 55 kDa band resulting from the early ER glycosylation, whereas the normal secreted M1 variant was also associated to the 58 kDa band corresponding to mature protein processed by Golgi apparatus (Figure S6).

### Interactions with ER Chaperones

**Figure 3:**
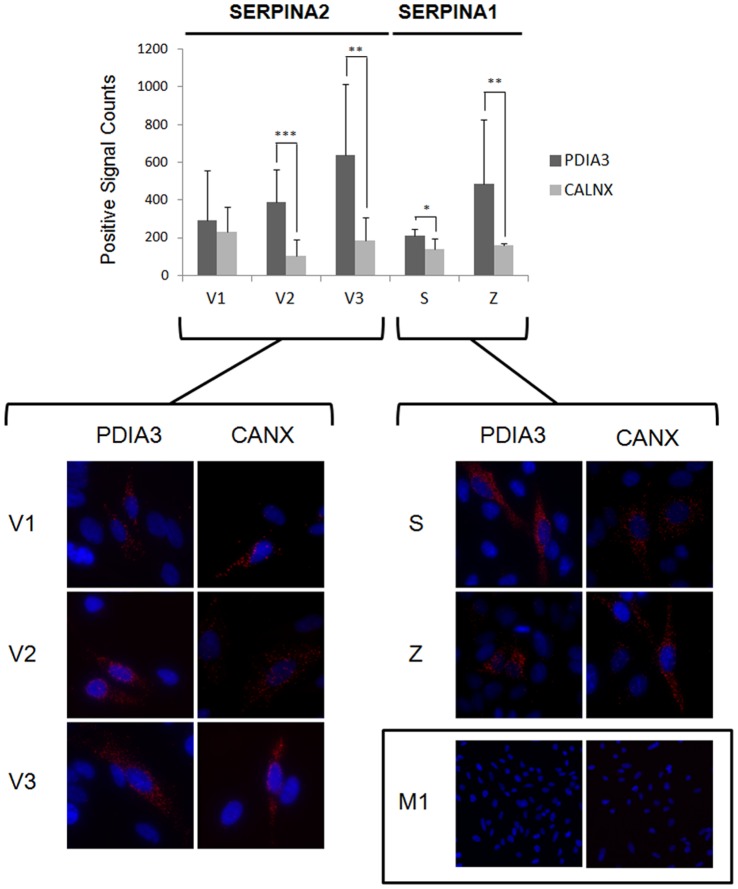
SERPINA1 and SERPINA2 interactions with ER chaperons PDIA3 and CANX. HeLa cells were stably transduced with *SERPINA2* (V1, V2 and V3) and *SERPINA1* (M1, S and Z) vectors. The interaction of SERPINs with PDIA3 and CANX was assessed by PLA. Cells were incubated with antibodies against V5 and PDIA3 or V5 and CANX. Close proximity of oligonucleotide-ligated secondary antibodies allowed the rolling-circle amplification and the detection of the rolling-circle amplification product by a fluorescently labeled probe. Nuclei were stained with DAPI. The number of spots per positive cell was quantified using Duolink image tool software. The graph presents the number of positive signals per cell; n = 3, mean+SD; *p<0.05; **p<0.01; ***p<0.001. Magnification 630×. The inset shows M1 negative results (Magnification 200×).

CANX and PDIA3 are chaperone molecules involved in the ER quality control of newly synthesized glycoproteins. To address if SERPINA2 and A1 variants directly interact with CANX and PDIA3 chaperones we performed PLA with antibodies against the V5-tag and CANX and against V5-tag and PDIA3 (Figure 3). Positive signals were detected in SERPINA2 variants and in S and Z, but not in M1. Both SERPINA2 and A1 present stronger interaction with PDIA3 chaperone, and this interaction is increased for variant V3 and Z (Figure 3). In all transduced HeLa cell lines the expression of SERPINA2 and A1 had no significant effect on CANX or PDIA3 protein levels as evaluated by Western Blot analysis (Figure S7).

### Degradation of Misfolded Proteins

**Figure 4:**
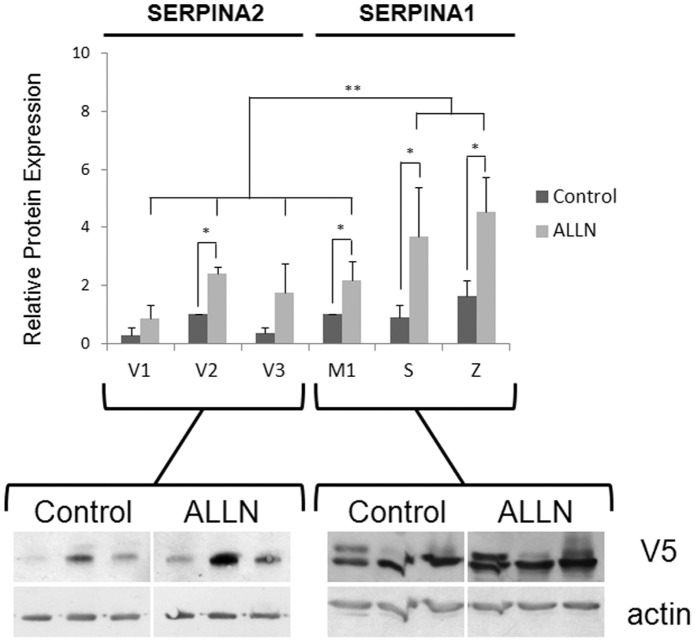
Analysis of the impact of proteasome inhibition in SERPINA2 and SERPINA1 expression. HeLa cells stably expressing SERPINA2 and A1 were treated with ALLN (50 µg/mL). Samples were subjected to 10% SDS-PAGE and immunoblotted for V5 tag. Actin was used as loading control. Relative protein expression was quantified through band intensity and presented graphically; n = 3, mean+SD; *p<0.05; **p<0.01.

The mechanisms of ER associated-degradation (ERAD) and autophagy have been implicated in the cellular clearance of different misfolded variants of SERPINs identified in the ER [18]–[21]. To determine whether proteolytic pathways regulate differently SERPINA2 and A1, stably transduced HeLa cells were treated with the proteasome inhibitor N-Acetyl-L-leucyl-L-leucyl-L-norleucinal (ALLN) and with the autophagy inhibitor 3-Methyladenine (3MA). In all SERPIN variants, the inhibition of the proteasome by ALLN led to increased levels of recombinant proteins (Figure 4). A significant increase of S and Z alleles was detected, while the non-deficient allele M1 and SERPINA2 were less affected and seemed regulated by the proteasome in a similar extent (Figure 4). On the other hand, autophagy inhibition with 3MA resulted in the accumulation of large protein granules in the Z variant, suggesting that it is degraded by autophagy and accordingly, accumulates in autophagosome structures. This is not the case for the other variants of SERPINA1 and A2 (Figure S8).

### Polymerization of Misfolded SERPINs and Heat Stress Response

**Figure 5:**
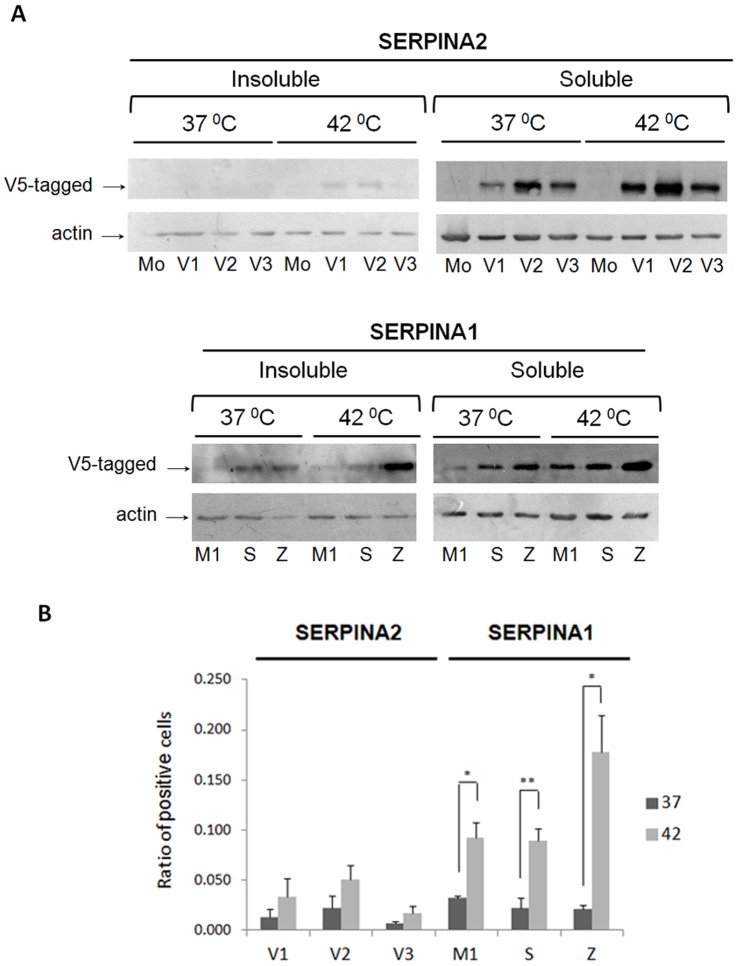
Protein polymerization and heat stress response. HeLa cells stably expressing SERPINA2 and A1 were incubated for 24 hours at 37 or 42 °C. **A** - Insoluble and Soluble protein extracts were separated by 10% SDS-PAGE. SERPINA2 and A1 were detected with anti-V5 and actin was used as loading control. **B** - Number of cells stained with V5 antibody were quantified and presented graphically; n = 3; mean+SD; *p<0.05; **p<0.01.

To evaluate the polymerogenic properties of SERPINA2 and A1 variants, we analyzed the insoluble fractions by Western Blot. Bands corresponding to polymerogenic SERPINs were detected in cells stably expressing S and Z. No bands were detected in the insoluble fraction of SERPINA2 (Figure 5A).

To induce the formation of SERPIN aggregates *in vitro*, all transduced cell lines were submitted to a heat stress (42°C) for periods of 24 hours. All variants showed an increment in protein expression as observed by immunoblot and immunofluorescence assays (Figure 5A and 5B). Upon heat stress, bands consistent with protein polymerization were detected in the insoluble fraction of all variants, including M1 and SERPINA2 variants. Increased expression was also observed in the soluble fraction of all variants analyzed when temperature sift was applied (42°C). Another effect of the heat stress was the increased number of cells expressing the recombinant SERPINs, which is only significant for the SERPINA1 variants.

### Tissue Expression Pattern of SERPINA2 and SERPINA1

**Figure 6:**
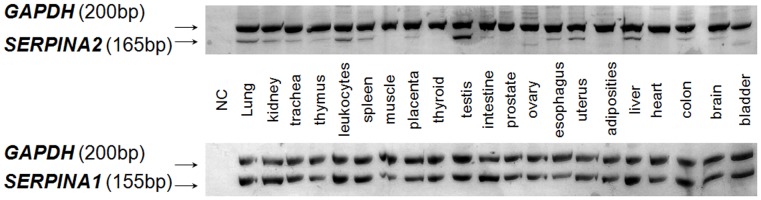
Expression of*SERPINA2* and *SERPINA1* in human tissues. Duplex PCRs carried out in a cDNA panel from human healthy organs, each one including a minimum of three donor’s pool. GAPDH amplification was used as internal control. NC – Negative Control.

To investigate *SERPINA2* expression a panel of 22 human tissues (cDNA) was screened by PCR. For comparison proposes, the expression of *SERPINA1* was also evaluated (Figure 6). Beside the expression in testes, leukocytes and liver, *SERPINA2* transcripts were also detected in brain, colon, uterus, esophagus, spleen, trachea, kidney and lung. *SERPINA1* showed a ubiquitous expression in the set of tissues tested, in strong agreement with the expressed sequence tag profile described for this gene (http://www.ncbi.nlm.nih.gov/UniGene/).

### *In vivo* Expression of *SERPINA2* and *SERPINA1*

**Figure 7:**
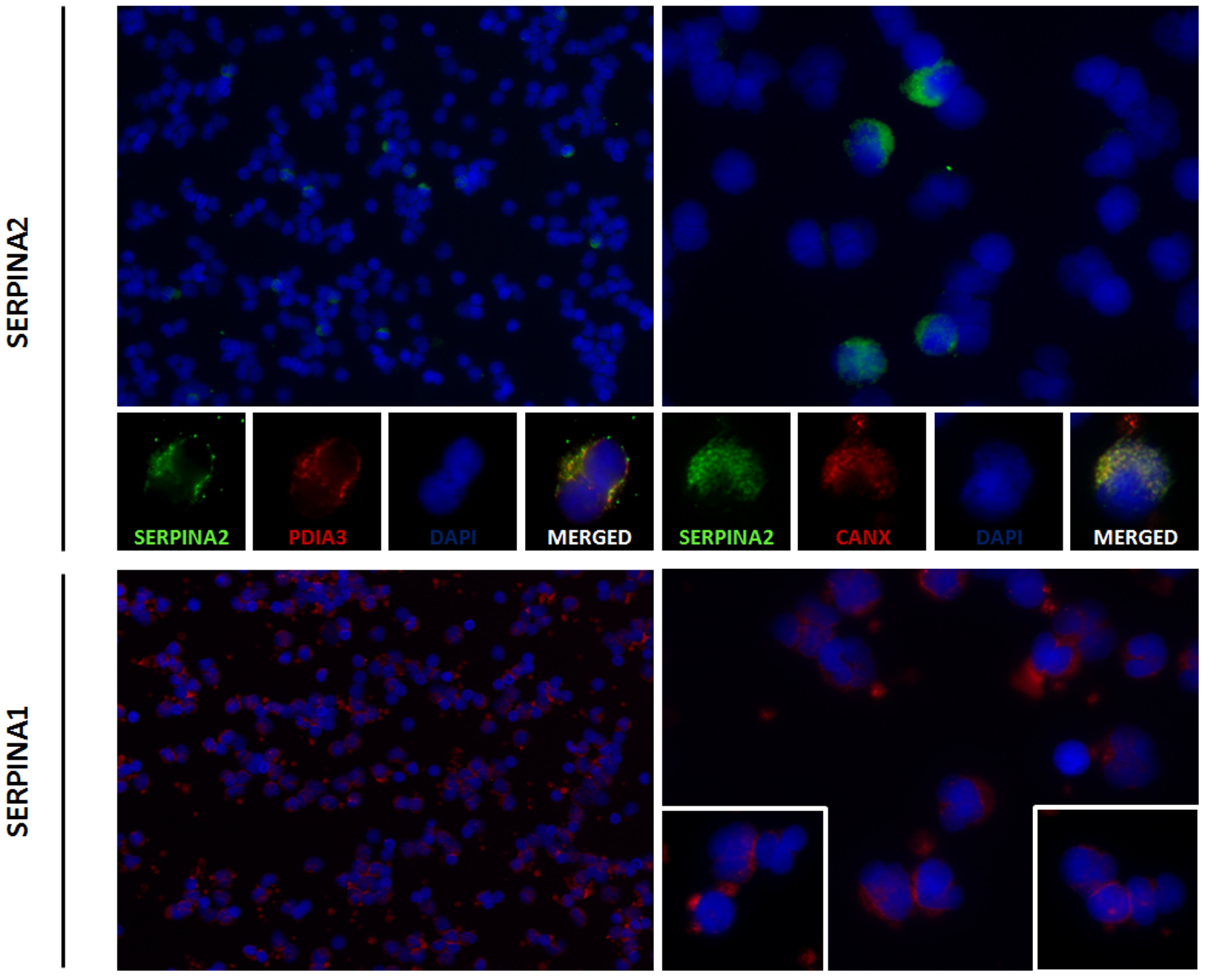
*In vivo* expression of SERPINA2 and SERPINA1. Leukocytes were collected from blood of an individual with a V2Null genotype for *SERPINA2* and a M1M2 genotype for *SERPINA1*. SERPINA2 was stained with K12 and Alexa Fluor 488 (green) antibodies. ER chaperones (CANX or PDIA3) were detected with anti-CANX or anti-PDIA3 and Alexa Fluor 594 (red) antibodies. SERPINA1 was stained with H203 and and Alexa Fluor 594 (red) antibodies. Nuclei were stained with DAPI. Magnification 200× (left panel) 630× (right panel and insets).

To validate the expression of SERPINA2 *in vivo* we carried out several assay in total leukocytes collected from blood. The results of the leukocytes staining with a monoclonal antibody against SERPINA2 demonstrate that the protein is translated *in vivo,* in a similar subcellular pattern to the observed in HeLa as confirmed by the co-localization with the ER chaperones CANX e PDIA3 (Figure 7 and Figure S9). The expression of SERPINA2 in a low percentage of leukocytes (∼8%) suggests that it may be specific of a subtype of these cells. Conversely, the expression of SERPINA1 is common to all leukocytes and it shows a different cellular distribution from SERPINA2, which may be attributed to the M1M2 genotype (Figure 7 and Figure S9).

### SERPINA2 and SERPINA1 Phylogenetic Analysis

**Figure 8:**
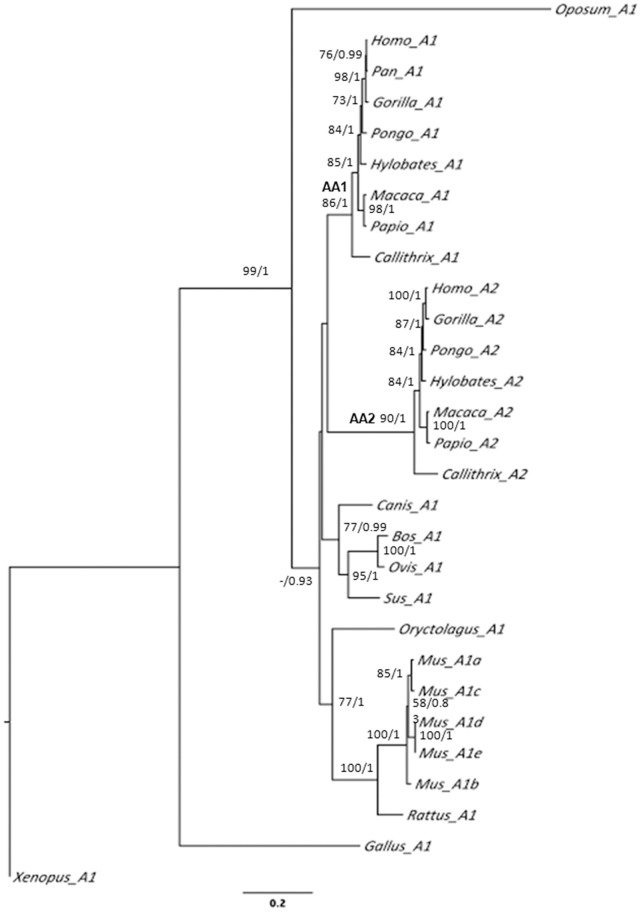
Maximum likelihood phylogenetic tree of *SERPINA2* and *SERPINA1*. Values shown by nodes are the bootstrap values and posterior probabilities obtained from the Bayesian Inference analysis. AA1– Ancestral *SERPINA1* branch for primates. AA2– Ancestral *SERPINA2* branch.

To reconstruct the evolutionary history of SERPINA2 and A1, we built a phylogenetic tree using a total of seven *SERPINA2* and 22 *SERPINA1* coding sequences (Table S1). The phylogenetic trees obtained by Maximum Likelihood (ML) and Bayesian Inference (BI) showed identical topologies (Figure 8). Four well-supported clades are observed within the placental mammals, which represent the Lagomorpha/Rodentia, Carnivora/Perissodactila and Primates; this latter group was subdivided into two clades representing the duplication of the *SERPINA1* and *A2*. Within these clades, the relationship between *SERPIN* sequences reproduces those of the species. However, the phylogenetic relationships between these clades are not well resolved, which is reflected in a basal polytomy in the BI tree and low bootstrap values in the ML tree (Figure 8). The ML phylogeny places the two primate’s SERPINs clades as sister groups, although there is no bootstrap supporting this sister relationship.

**Figure 9:**
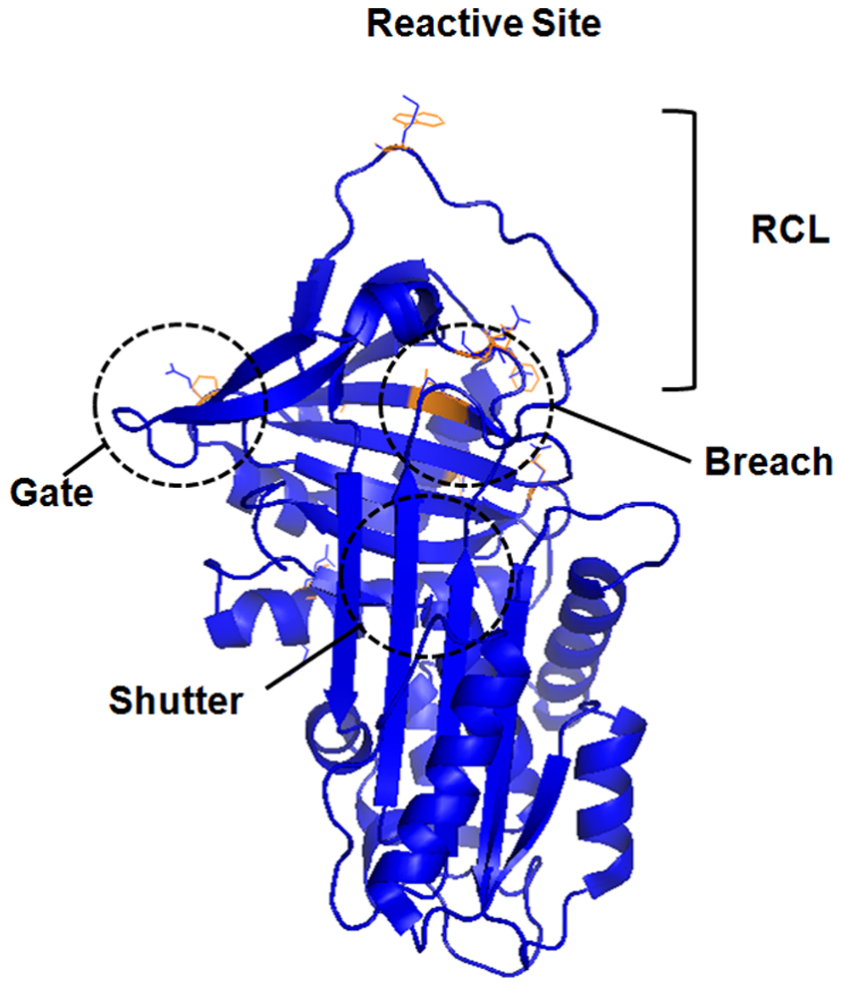
Overlapped tridimensional model of SERPINA1 and SERPINA2. The model was built using SERPINA1 structure (pdb code: 1QLP) and SERPINA2 theoretical model [17]. The image indicates the most important regions for inhibitory function – RCL, breach, shutter, and gate. Amino acids substitutions favored by selection are highlighted in orange. This figure was generated using PyMOL (PyMOL. DeLano Scientific, San Carlos) [54].

In order to measure and characterize the selective pressure operating on the *SERPINA1* and *A2*, we calculated *d_N_*/*d_S_* (ω; *d_S_* – synonymous substitution rate, and *d_N_* - non-synonymous substitution rate) ratios for the entire phylogeny (M0 model) and for all tree branches (free-ratio model) [22]. Although our results suggest an overall conserved evolution as indicated by the ω value below one (0.34), the higher likelihood obtained for all tree branches (free-ratio model) suggest that different lineages might have experienced different evolutionary rates [*branch*-model (M0– free): −2Δl = 167.39; P<0.001). Here, the inclusion of highly divergent sequences (*Xenopus*, *Gallus* and *Monodelphis*) is likely to have influenced our results, given the unrealistic values (ω = ∞ or *d_S_* = 0) estimated for the more ancestral branches. In a different approach, we used the primate clade for each gene alone to address the evolution of the two homologues. According to the estimated ω values (*SERPINA1* ω = 0.49 and *SERPINA2* ω = 0.56) both genes are likely to be conserved. Importantly, however, the evolutionary rates displayed by ancestral branches of *SERPINA2* and *A1* (AA2 and AA1 branches, respectively; Figure 8) were quite dissimilar and consistent with the differences observed in the tree branch length. While the ancestral *SERPINA1* branch (AA1) yielded low values (ω = 0.32; *d_N_* = 0.04 and *d_S_* = 0.14), *SERPINA2* ancestral branch (AA2) presented much higher scores (ω = 0.59; *d_N_* = 0.20 and *d_S_* = 0.33) pointing to a possible relaxation of selective constraints after duplication. Therefore, to test whether *SERPINA2* could have undergone a process of adaptive evolution, we performed the *branch-sites* model test [23], [24]. Even though the vast majority of sites are constrained or neutrally evolving, 11 amino acid positions show a signature of positive selection in *SERPINA2* with a posterior probability higher than 80% (ω = 9.08; P<0.01) (Table 1). From the analysis of the comparative structural model of SERPINA1 and A2, the codons with higher probabilities of being selected are located in the reactive site (M358W), in the breach (R196D; P197K; L241A; M242Q), and spread over other regions of the molecule (R39K; C232D; E257P T273E; E279F and Q377D; Figure 9). According to the predictions of Polyphen [25], [26], and focusing on the crystallographic structure of SERPINA1, only two of the positively selected SERPINA2 aminoacids are expected to affect the protein (L241A; M242Q; Table S2).

**Table 1:** Phylogenetic-based test of*SERPINA2* diversification.

*Branch-site* model (−2Δ*l* = 19.77; P<0.01)				
Site classes^a^	0	1	2a	2b
Neutral Hypothesis: Sites are under purifying selection or neutrally evolving. *l* = −4940.50				
Proportions	0.49	0.39	0.07	0.05
Background ω	0.14	1.00	0.14	1.00
Foreground^b^ ω	0.14	1.00	1.00	1.00
Selective Hypothesis: Several sites are positively selected *l* = −4930.62				
Proportions	0.53	0.35	0.07	0.05
Background ω	0.18	1.00	0.18	1.00
Foreground^b^ ω	0.18	1.00	9.08	9.08
Positively selected sites (Posterior Probabilities): R39K; R196D; P197K; C232D; L241A; M242Q; E257P; T273E; E279F M358W Q377D (0.94;0.92;0.97; 0.86; 0.83;0.85; 0.83; 0.82; 0.89; 0.89; 0.81).				

a Sites Classes: 0– sites under constrains; 1– neutral sites; 2a – constrained sites under positive selection in the *foreground* branch; 2b – neutral sites under positive selection in the *foreground* branch.

b *foreground* branch – ancestral *SERPINA2* branch (AA2).

## Discussion

In this study, we perform a comparative analysis for *SERPINA2* and *A1*, which share the highest level of sequence identity amongst genes of the 14q32.1 *SERPIN* cluster, and were thought to represent an inactivated duplicate and its functional gene, respectively. We cloned and sequenced three major full transcripts of *SERPINA2* differing at 308 and 320 codon positions and confirmed the gene organization in four coding exons, as initially predicted from alignments with *SERPINA1* [15]. Furthermore, we provide the first *in vitro* and *in vivo* evidence for the translation of SERPINA2 into a stable protein, modified by the addition of N-linked glycosyl side chains, with a molecular weight of ∼52 kDa (without V5-tag) compatible with a regular SERPIN.

However, in contrast to SERPINA1, which is processed into a mature protein (∼55 kDa) by the Golgi apparatus to include complex side chains resistant to Endo H, SERPINA2 is only partially glycosylated and sensitive to Endo H, suggesting a subcellular localization in the ER. Indeed, the reticular pattern observed for SERPINA2, the subcellular fractionation into the membranous fraction, and the co-localization with CANX and PDIA3, two chaperones involved in the ER quality control of newly synthesized glycoproteins provided additional support for SERPINA2 localization in this organelle. Moreover, SERPINA2 is absent in the extracellular media, suggesting that its localization to the ER is complete, in contrast to S and Z alleles of SERPINA1, which are partially secreted despite their abnormal accumulation in the ER due to misfolding.

CANX has a more prolonged association to misfolded proteins entering in the ERAD pathway and has been implicated in the degradation of Z variant [27]–[31]. In our models, only a minor fraction of the recombinant proteins Z and S, and SERPINA2, associate with CANX as revealed by PLA, and no significant difference is observed among variants or between SERPINA1 and A2. Only M1 does not bind to CANX. This shows that all variants are in close proximity (maximum 30–40 nm) to CANX, supposedly as a result of protein-protein interaction. In the case of the misfolded Z variant of SERPINA1, dissociation from CAXN is essential for its rapid degradation by the proteasome, and prolonged interactions may imply an impaired function of the ER [31], providing an alternative justification for the lack of distinction between SERPINs.

The PDIA3 chaperone catalyses the biochemical reactions of proteins entering in the CANX cycle and is recruited by unfolded proteins in aggregates [32]–[34], presenting a stronger association with SERPINs than CANX. These results are in agreement with the previous findings on Z transgenic mice in which a protein-disulfide isomerase (P4hb or Pdi) exhibited a more permanent association to Z, probably favoring protein unfolding and delivery to the proteasome pathway [35]. Importantly, P4HB was found to co-localize with Z in the ER and in the inclusion bodies [31], and contrary to CANX, the overexpression of P4HB and PDIA3 did not affect ER trafficking or degradation of misfolded SERPINs [31], [36]. Proteasome inhibition with ALLN in SERPINA2 cell lines contrasts with the features of SERPINA1 variants, significantly degraded by this proteolytic pathway. SERPINA2 variants present residual levels of proteasome-dependent degradation, comparable to the M1 variant of SERPINA1. Nevertheless, it remains unclear whether the degradation of SERPINA2 could be attributed to abnormal protein folding. We hypothesized that other proteolytic pathways could contribute on the regulation of SERPINA2, such as autophagy, similarly to that described for Z variant [18], [28]–[31], [37], [38]. Interestingly, in our models, the inhibition of authophagy by 3MA was found to result in the accumulation of Z in autophagosome-like structures, but this was not observed for S and SERPINA2 variants.

Conversely, the identification of insoluble fractions for S and Z but not for SERPINA2, which was only present in the soluble fraction, suggests that SERPINA2 may be a non-polymerogenic SERPIN. Our results suggest that SERPINA2 resides in the ER without being misfolded, as shown by the insensitivity to proteasome or autophagy inhibition. Interestingly, other SERPINs have been shown to reside and participate in chaperone-assembly at the ER. For example SERPINH1, also known as HSP47, is an ER-resident molecule enrolled in the biosynthesis of collagen, that interacts with different ER chaperones possibly through a large protein complex [39], [40]. For the case of SERPINA2, our results show that it interacts directly with CAXN and PDIA3, either as a substrate or as a co-chaperone. Despite the absence of the RDEL (SERPINH1) or the KDEL C-terminal motif that provides an ER anchor, SERPINA2 is predicted by WoLF PSORT program [41] to have ER localization (14/32 neighbors). The fact that the extracellular prediction (10/32 neighbors) might be strongly influenced by common domains of clade A SERPINs (7 of the 10 neighbors), indicates that the ER localization of SERPINA2 is likely, and suggests the presence of unknown ER motifs in this protein.

Temperature increments have been widely proposed to favor Z polymerization *in vivo* and *in vitro* [6], [42]. In our models, heat stress had a minor impact in SERPINA2 and a higher effect in SERPINA1, as seen by the levels of recombinant proteins found in insoluble fractions. In contrast, SERPINA2 showed only a slight increase in its expression reinforcing the hypothesis of a divergent activity from SERPINA1.

The phylogenetic analysis of *SERPINA1* and *A2* sequences placed the duplication event of these genes in the crown of Primate group, about 92.7 million years ago [43]. The lines of evidence provided by our phylogenetic analysis are consistent with a constrained evolution of *SERPINA2* and *A1* in primates (*d_N_*/*d_S_* <1), thereby sustaining the hypothesis of *SERPINA2* being an active gene, expressed in a large set of tissues and coding for a stable SERPIN. Nonetheless, the finding of a higher evolutionary rate in *SERPINA2* ancestral branch (AA2) and the evidence for several amino acid substitutions being evolving under positive selection, support the hypothesis of the emergence of a new advantageous function after duplication. These substitutions include the M358W in the reactive site, and the L241A and M242Q in the second strand from B β-sheets (s2B). In *in vitro* assays carried out in a SERPINA1 scaffold, the M358W substitution was shown to slower the formation of protease-inhibitor complexes and to change the inhibitory affinity towards chymotrypsin [44]. On the other hand, in the three-dimensional models of SERPINA1 the L241A and M242Q substitutions are predicted to create a cavity and to reduce hydrophobicity at buried sites of the protein, respectively. Moreover, these sites are expected to pack against W194 and F198, two conserved residues among the entire SERPIN superfamily [3]. In close proximity to the aforementioned residues there are two other substitutions (R196D and P197K) inferred as targets of positive selection in SERPINA2. Interestingly, all these residues lie in a key region of SERPIN’s structure – the breach, in which significant changes in composition were shown to affect the “stressed” to “relaxed” transition and to increase protein stability with a concomitant decrease in the inhibitory activity [45]–[48].

Considering the potential effects of the amino acid substitutions favored by selection, we propose that SERPINA2 has evolved toward a different protease affinity and an altered plasticity. The most recent advances in the understanding of SERPIN misfolding indicate the swapping of the protein C-terminus region into a B β-sheet gap of another molecule (s4B and s5B) as the underlying cause for the pathological polymerization of the Z allele in vivo [49], [50]. The finding of a reduced susceptibility to polymerization by SERPINA2 might suggest a significant impact of selected residues in maintaining a more stable conformation and a structure less prone to C-terminus swapping.

In summary, herein we provide the first *in vitro*, *in vivo,* and phylogenetic evidences for *SERPINA2* as an active gene and not genomic redundant. We show that SERPINA2 is an intracellular glycoprotein with an ER localization that cannot be associated to protein polymerization or misfolding. Moreover, we demonstrated that *SERPINA2* has undergone an accelerated divergence from *SERPINA1*, which contributed to the acquisition of a new activity, possibly as a chymotrypsin-like SERPIN conserved in most present-day primates.

## Materials and Methods

### cDNA Isolation and Cloning of SERPINA2 and SERPINA1

The cDNA corresponding to *SERPINA2* full transcript (without ATA and 2 kb deletion) was amplified from a human testes cDNA library (Clontech) using specific primers (Fw: 5′- CAC CAT GCC ATT CTC TGT CTC ATG -3′; Rv: 5′- TTT TTG GGT GGG ATT CAC CAC T -3′). The cDNA corresponding to *SERPINA1* was amplified from leukocyte total mRNA of two individuals with known genotypes (M1S and M3Z), using specific primers (Fw: 5′-CAC CAT GCC GTC TTC TGT CTC GTG GGG CA-3′ Rv: 5′ TTT TTG GGT GGG ATT CAC CAC T -3′). The amplicons from *SERPINA2* and *A1* were cloned into a pLenti6/V5 vector (Life Technologies) and sequenced. Clones containing distinct *SERPINA2* and *A1* variants were selected for downstream experiments.

### Establishment of SERPINA1 and SERPINA2 Stable Cell Lines

293FT (Life Technologies) and HeLa (ATCC number CCL-2) cells were grown in Dulbecco's Modified Eagle Medium (DMEM; Life Technologies) containing 10% fetal bovine serum (FBS; Life Technologies) and penicillin/streptomycin antibiotics. Each vector was co-transfected into 293FT with ViraPower Packaging Mix (Life Technologies) at a 1∶1 ratio using lipofectamine agent (Life Technologies). The cell media containing the lentivirus particles was collected 48 and 72 hours after, and used to transduce HeLa cell lines. Selection of positive cells, stably expressing SERPINA2 or A1 was carried out for 1 week using 10 mg/ml of blasticidin (Life Technologies). In addition HeLa cells were transduced with a pLenti6/V5 empty vector (mock) which was used as negative control.

### Protein Extraction and Western Blot

The intracellular protein extracts were obtained by scraping cells with cold Catenin lysis buffer –1% Triton X-100 (Sigma), 1% Nonidet P-40 (Sigma) in PBS – enriched with protease inhibitor (Roche) and phosphatase inhibitor (Sigma) cocktails. Cells were centrifuged at 12000 g for 20 min at 4°C and the pellets were sonicated in 50 µl of buffer containing 60 mM Tris-HCl, pH 6.8, 5% SDS and 10% glycerol, to recover the proteins in the insoluble fraction. The extracellular protein extracts were obtained by maintaining cells in conditioned media (DMEM with antibiotics only) for 24 hours before media harvesting. The media was desiccated by vacuum and re-suspended in 0.1% tricholoroeacetic acid. The total protein concentration was determined by Bio-Rad protein assay kit II (Bio-Rad). Proteins (∼50 µg) were mixed with 4×gel loading buffer (4% SDS, 20% glycerol, 120 mM Tris-HCl, pH 6.8, 0.01% bromophenol blue, 2% β-mercaptoethanol), heated to 95°C for 3–5 min, and separated by SDS-PAGE (10%–12% poly-acrylamide). For the immunodetection of recombinant SERPINs, the separated proteins were transferred into a nitrocellulose membrane (GE Healthcare), this was blocked in PBS-T (0.5% Tween-20) containing 5% non-fat milk, and probed with the primary antibodies against V5-tag (Life Technologies), actin (Sigma), CANX (Enzo Life Sciences) and PDIA3 (Sigma). Immunoblots were visualized using ECL detection kit (GE Healthcare). Intracellular proteins were enzymatically deglycosylated with Endo H (Roche) and NGF (Roche) according to manufacture instructions.

### Immunocytochemistry

Cells were grown in coverslips, fixed in ice-cold methanol, blocked with a 2% *bovine serum albumin* solution for 30 minutes, and incubated overnight at 4°C with the following primary antibodies, diluted at a 1∶200 ratio: anti-V5, anti-CANX, anti-PDIA3 and anti-SERPINA1 (H203; Santa Cruz Biotechnology, inc); or diluted at 1∶100 ratio: anti-SERPINA2 (K12; Santa Cruz Biotechnology, inc). For fluorescence imaging, coverslips were incubated with the appropriate secondary antibodies, diluted at a 1∶500 ratio, Alexa Fluor 488-labeled antibody (Life Technologies) or Alexa Fluor 594-labeled antibody (Life Technologies) and counterstained using Vectashield with DAPI (Vector Laboratories). Images were acquired in a Carl Zeiss Apotome Axiovert 200 M Fluorescence Microscope and a Leika TCS SP5 Confocal Microscope.

### Subcellular Protein Fractionation

To determine the subcellular localization of SERPINA2 and SERPINA1 variants, transduced HeLa cells (1×10^6^ cells) were harvested and fractionated into different subcellular extracts using the subcellular protein fractionation kit (Thermo Scientific) according to the manufacturer’s protocol. The total content from the membranous and cytoplasmic fractions was precipitated with acetone, separated by SDS-PAGE and analyzed by Western Blot with the primary antibodies against V5-tag (Life Technologies) and KDEL (Enzo Life Sciences).

### Proximity Ligation Assay (PLA)

To test for possible protein-protein interactions with ER chaperones, we used PLA analysis using Duolink kit (Olink Bioscience) according to the manufacturer’s instructions. Briefly, cells were grown in coverslips, fixed in ice-cold methanol, blocked with the Duolink blocking solution and incubated with same antibodies, as for the imunocytochemistry. Afterwards, coverslips were incubated with the secondary antibodies linked to the PLA probes and specific oligonucleotides were hybridized to PLA probes and circularized by ligation. The DNA circle was then amplified using rolling circle amplification into a bundle of single stranded DNA anchored to one of the antibodies, which could be detected by the addition of complementary fluorophore-labeled oligonucleotides. Images were acquired in a Carl Zeiss Apotome Axiovert 200 M Fluorescence Microscope and quantification of positive signals in 3 or more fields was carried out with Duolink image tool software (Olink Bioscience).

### Cell Stress Induction Assays

To assess the effect of the heat stress cells were incubated for 24 hours at 42°C. The response to stress inducers was evaluated by immunocytochemistry and immunoblotting. To assess the effect of proteasome inhibition we used ALLN (Sigma) and 3-Methyladenine (3MA; Sigma) was used to inhibit autophagy. Cells were seeded on 6-well plates, and treated for 18 hours with ALLN (50 µg/mL), or for 24 hours with 3MA (10 µM).

### Tissue Expression Screening of SERPINA2 and SERPINA1

To investigate the pattern of distribution of *SERPINA2* and *A1* transcripts we analyzed 22 cDNA samples from different healthy organs. Except for the first-strand cDNA from leukocytes (Clontech), the tissue cDNA samples were synthesized by reverse transcriptase methods using as templates the RNA from the First Choice Human Total RNA Survey Panel (Ambiom). Reverse transcription was performed using the Superscript III RT PCR system (Life Technologies) according to the manufacturer’s protocol. The primers 5′- GGC TGA TCT ATC ACA AAC CA -3′ and 5′- AAG CAT TCG TGG ATC TTG GC -3′ were used for the amplification of *SERPINA2* and the primers 5′- CAA TGG CCT GTT CCT CAG C-3′ and 5′- CTT GAG TAC CCT TCT CCA CG-3′ were used for the amplification of *SERPINA1* cDNA. The amplification of a segment from *GAPDH* was employed as internal control using the primers 5′-TCA AGG CTG AGA ACG GGA AG -3′ and 5′-AGA GGG GGC AGA GAT GAT GA-3′.

### *In vivo* Expression

Leukocytes were isolated from a blood of an individual with a V2Null genotype for *SERPINA2* and with a M1M2 genotype for *SERPINA1*. Leukocytes were collected by centrifugation at 2000 g for 5 min at 4°C after the lysis of erythrocytes with a commercial buffer (CitoMed). The leukocytes pellet was rinsed in 2% paraformaldehyde and submitted to cytospin preparation. Immunocytochemistry of leukocyte preparations were performed as described above.

#### Statistical analysis

Data are presented as means with standard deviations. Statistical analysis was performed by means of the t-test or ANOVA. Values of P<0.05 were considered significant.

### Divergence of*SERPINA2* and *SERPINA1* Orthologues

*SERPINA2* and *A1* cDNA sequences were retrieved from available databases or obtained by direct sequencing (Table S1). Phylogenetic analyses of the 29 vertebrate sequences were performed using ML in PAUP* v4.0b10 and BI in MrBayes 3.2 [51]. Heuristic searches were run with ML as optimality criterion. The starting tree was obtained via stepwise addition, with 100 replicates of random addition of sequences. Tree-bisection-reconnection was used as the branch-swapping algorithm. The models of evolution implemented in the ML analyses were obtained with the Akaike Information Criterion (AIC) implemented in jModelTest 0.1.1 [52]. To test the robustness of the trees 1000 bootstrap replicates were run. Bayesian analyses were run using the model of evolution determined with jModelTest, and parameters were incorporated as priors in the form of dirichlet distributions. Two independent runs of 1×10^6^ generations with four chains each (1 cold and 3 heated chains) were set up. Trees were sampled every 100^th^ generation and the first 2500 trees were discarded as “burn-in”. The remaining trees were used to compute the Bayesian posterior probabilities of each clade of the consensus tree. *Serpina1* sequences of *Gallus* and *Xenopus* were used as outgroups.

Maximum likelihoods estimates of *d_N_/d_S_* (*ω*), where *d_S_* and *d_N_* correspond to synonymous and non-synonymous substitution rates respectively, were carried out using the *codeml* program from the software package Phylogenetic Analysis by Maximum Likelihood - PAML version 4.2 [53]. Likelihood-ratio tests (LRT) were performed using either the entire phylogeny or the primate clades as inputs. These included the *branch* model, which compares a single ω value obtained for all lineages (M0) with a model assuming different ω values for each lineage or branch (free-ratio) [22]. The values of ω >1 were considered as evidences of positive selection and the values of ω <1 were regarded as an indication of purifying selection. The significance of each nested model was obtained from twice the variation of likelihoods (2Δl) using a χ^2^ statistic. To evaluate whether the duplication event leading to *SERPINA2* could have been associated with a diversifying process, we performed the *branch-site* model for the primate clade including *SERPINA2* and *A1*. This model assumes that the branches on the phylogeny are divided a priori into *foreground* and *background* and allows ω to vary both among sites in the protein and across branches. For the *branch-site* model, comparisons with critical χ^2^ were carried out as described [23], [24]. The Bayes empirical Bayes (BEB) was used to calculate posterior probabilities of site classes, in order to identify sites under positive selection for the significant LRTs [23], [24].

## Supporting Information

Figure S1 **SERPINA1 and SERPINA2 sequences.** Alignment of SERPINA1 and SERPINA2 proteins, SERPINA2 was inferred from cDNA testes library sequencing. SERPINA1 (NM_000295.4) and SERPINA2 (JX680599) were used as references. Alignments were carried out by ClustalW implemented in MEGA5 software (http://www.megasoftware.net/). Variable sites and highlighted in blue and signal peptide in red.
(TIF)

Figure S2 **Expression of** ***SERPINA2*** **and** ***SERPINA1*** **in HeLa cells.** Duplex PCR carried out in cDNA derived from HeLa cells stably transduced with the empty vector (Mo) and *SERPINA2* (V1, V2 and V3) and *SERPINA1* (M1, S and Z) vectors. GAPDH amplification was used as internal control. NC –Negative Control. MW – Molecular Weight.
(TIF)

Figure S3 **Subcellular localization of SERPINA2 and SERPINA1 variants.** HeLa cells were stably transduced with *SERPINA2* (V1, V2 and V3) and *SERPINA1* (M1 and Z) vectors. SERPINA2 and A1 were stained with V5 and Alexa Fluor 488 (green) antibodies. ER chaperons (CANX or PDIA3) were detected with CANX or PDIA3 and Alexa Fluor 594 (red) antibodies. Nuclei were stained with DAPI. Magnification 400× (confocal microscopy; z-stacks).
(TIF)

Figure S4 **SERPINA2 expression in CHO cells.** CHO cells (ATCC number CCL-61) were stably transduced with *SERPINA2* (V1, V2 and V3) vectors. SERPINA2 was stained with anti-V5 and Alexa Fluor 488 (green) antibodies. Nuclei were stained with DAPI. Magnification 1000×.
(TIF)

Figure S5 **Co-localization of SERPINA1 with the Golgi apparatus.** HeLa cells were stably transduced with *SERPINA1* (M1, S, and Z) vectors. SERPINA1 was stained with H203 and Alexa Fluor 594 (red) antibodies. Golgi apparatus was detected with GM130 (BD Biosciences) and antibodies Alexa Fluor 488 (green) antibodies. Nuclei were stained with DAPI. Magnification 630×.
(TIF)

Figure S6 **Subcellular protein fractionation of SERPINA2 and SERPINA1**. HeLa cells were stably transduced with *SERPINA2* (V1, V2 and V3) and *SERPINA1* (M1, S and Z) vectors. Membranous and cytoplasmatic fractions were separated by 10% SDS-PAGE. ER protein (KDEL) was detected with anti-KDEL antibody. SERPINA2 and A1 proteins were detected with anti-V5 antibody.
(TIF)

Figure S7 **Effect of SERPINA2 and SERPINA1 expression in ER chaperons.** HeLa cells were stably transduced with the empty vector (Mo) and with *SERPINA2* (V1, V2 and V3) and *SERPINA1* (M1, S and Z) vectors. Intracellular lysates were separated by 10% SDS-PAGE. ER chaperones were detected with anti-CANX or anti-PDIA3 antibodies. The other proteins were detected with anti-V5 and anti-actin antibodies.
(TIF)

Figure S8 **Impact of autophagy inhibition in the expression SERPINA2 and SERPINA1.** **A -** HeLa cells were treated with 10 µM of 3-MA for 24 hours. SERPINA2 and A1 were stained with V5 and Alexa Fluor 488 (green) antibodies. Nuclei were stained with DAPI. Magnification 1000×. **B -** Intracellular lysates were separated by 10% SDS-PAGE. Proteins were detected with anti-V5, anti-p62 (Santa Cruz Biothecnology) and anti-actin antibodies. The accumulation of the autophagic substrate p62 confirms the inhibition of autophagic response in 3-MA treated cells. The intensity of the bands was normalized against actin and the non-treated sample.
(TIF)

Figure S9 **SERPINA2 and SERPINA1 expression in leukocytes and HeLa cells.** Leukocytes collected from blood of an individual with a V2Null genotype for *SERPINA2* and a M1M2 genotype for *SERPINA1*. HeLa cells were stably transduced with SERPINA2 (V1, V2 and V3) and SERPINA1 (M1, S and Z) vectors. SERPINA2 was stained with K12 and Alexa Fluor 488 (green) antibodies. SERPINA1 was stained with H203 and and Alexa Fluor 594 (red) antibodies. Nuclei were stained with DAPI. Magnification 630×.
(TIF)

Table S1 **Accession numbers of sequences used in the phylogenetic analysis.**
(DOCX)

Table S2 **Polyphen predictions and scores based on SERPINA1 structure for residues with higher probabilities of being positively selected during** ***SERPINA2*** **divergence.**
(DOCX)
