# Supplementary figures and images for: SERPINA2 Is a Novel Gene with a Divergent Function from SERPINA1
Source: PLoS One. 2013 Jun 24;8(6):e66889. doi: 10.1371/journal.pone.0066889 (PMC3691238; doi:10.1371/journal.pone.0066889)

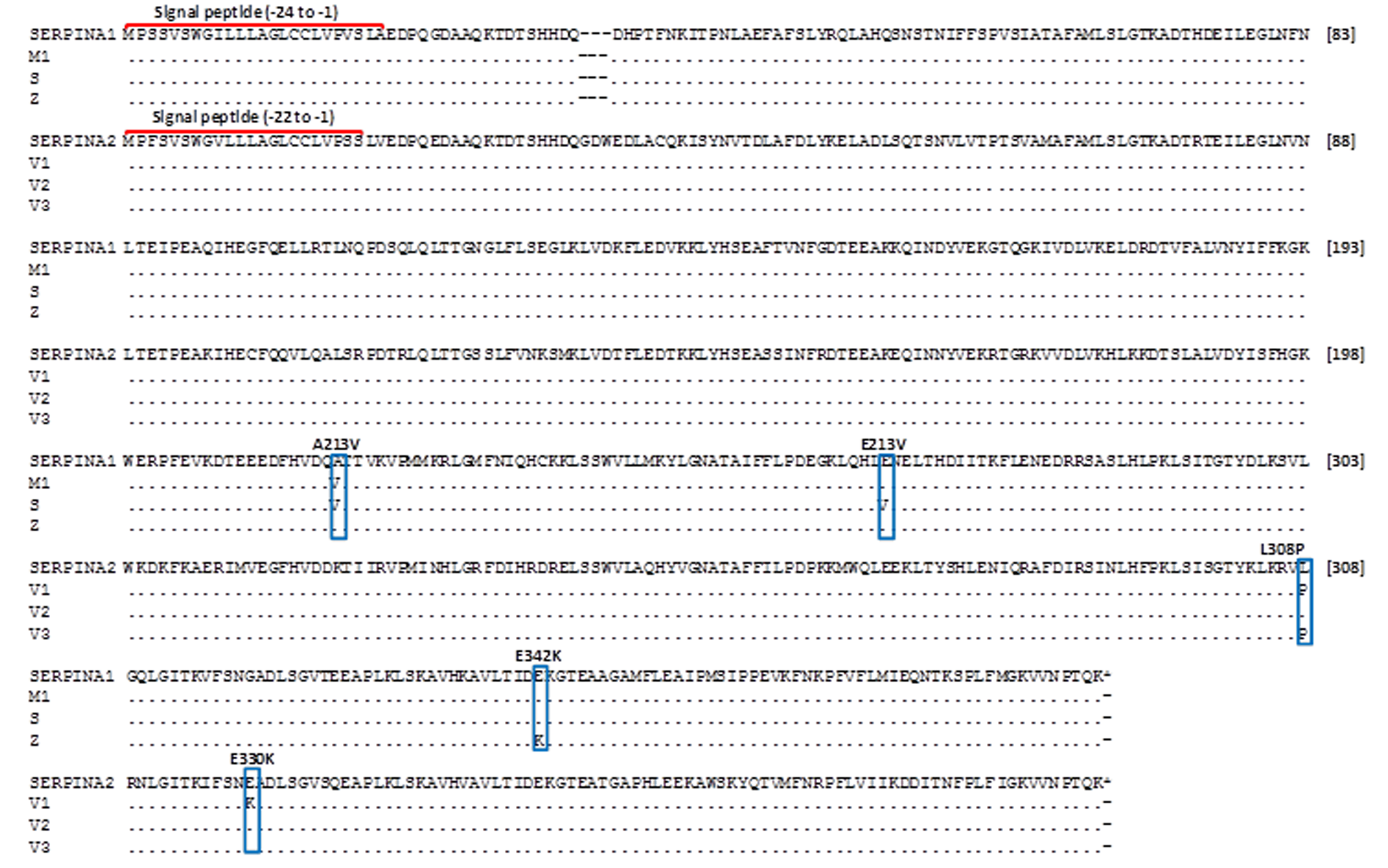

Supplement: Figure S1 — SERPINA1 and SERPINA2 sequences. Alignment of SERPINA1 and SERPINA2 proteins, SERPINA2 was inferred from cDNA testes library sequencing. SERPINA1 (NM_000295.4) and SERPINA2 (JX680599) were used as references. Alignments were carried out by ClustalW implemented in MEGA5 software (http://www.megasoftware.net/). Variable sites and highlighted in blue and signal peptide in red. (TIF) [file pone.0066889.s001.tif]

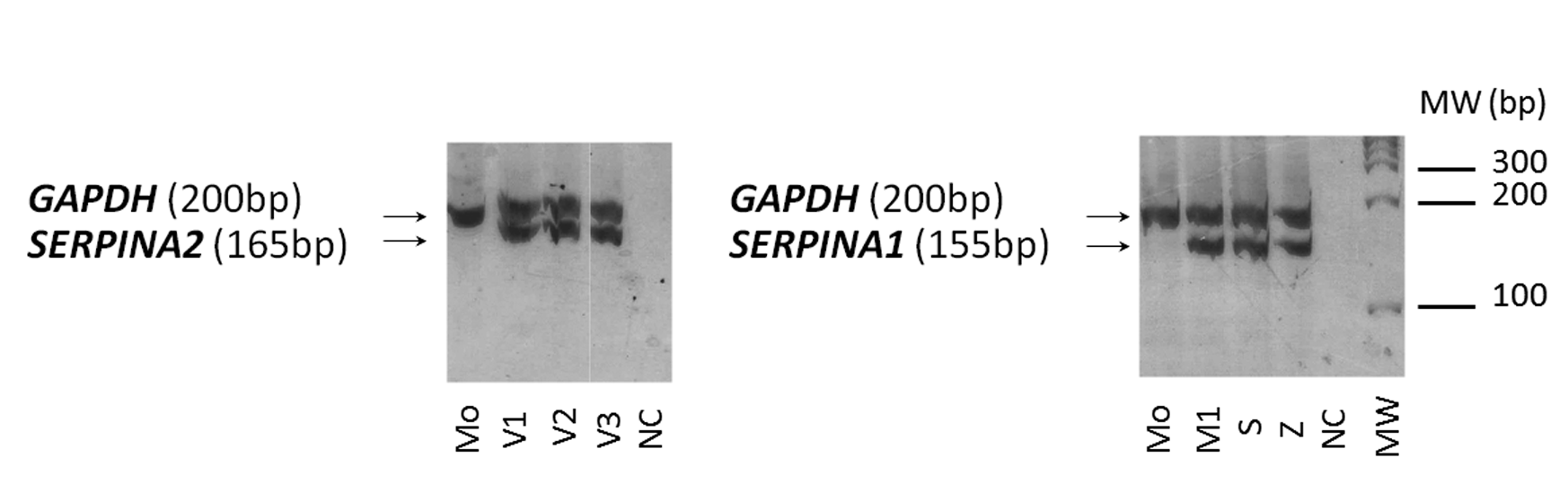

Supplement: Figure S2 — Expression of SERPINA2 and SERPINA1 in HeLa cells. Duplex PCR carried out in cDNA derived from HeLa cells stably transduced with the empty vector (Mo) and SERPINA2 (V1, V2 and V3) and SERPINA1 (M1, S and Z) vectors. GAPDH amplification was used as internal control. NC –Negative Control. MW – Molecular Weight. (TIF) [file pone.0066889.s002.tif]

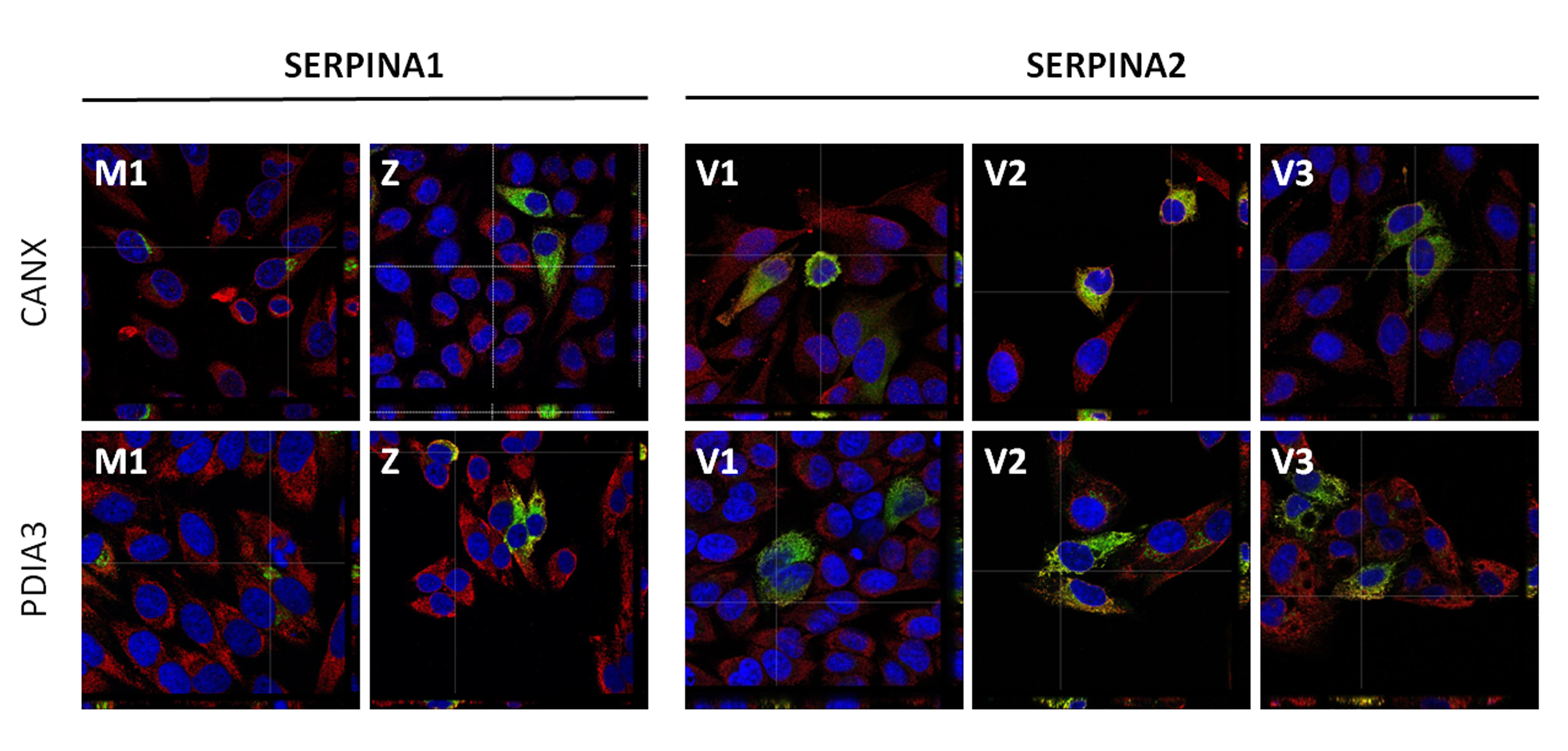

Supplement: Figure S3 — Subcellular localization of SERPINA2 and SERPINA1 variants. HeLa cells were stably transduced with SERPINA2 (V1, V2 and V3) and SERPINA1 (M1 and Z) vectors. SERPINA2 and A1 were stained with V5 and Alexa Fluor 488 (green) antibodies. ER chaperons (CANX or PDIA3) were detected with CANX or PDIA3 and Alexa Fluor 594 (red) antibodies. Nuclei were stained with DAPI. Magnification 400× (confocal microscopy; z-stacks). (TIF) [file pone.0066889.s003.tif]

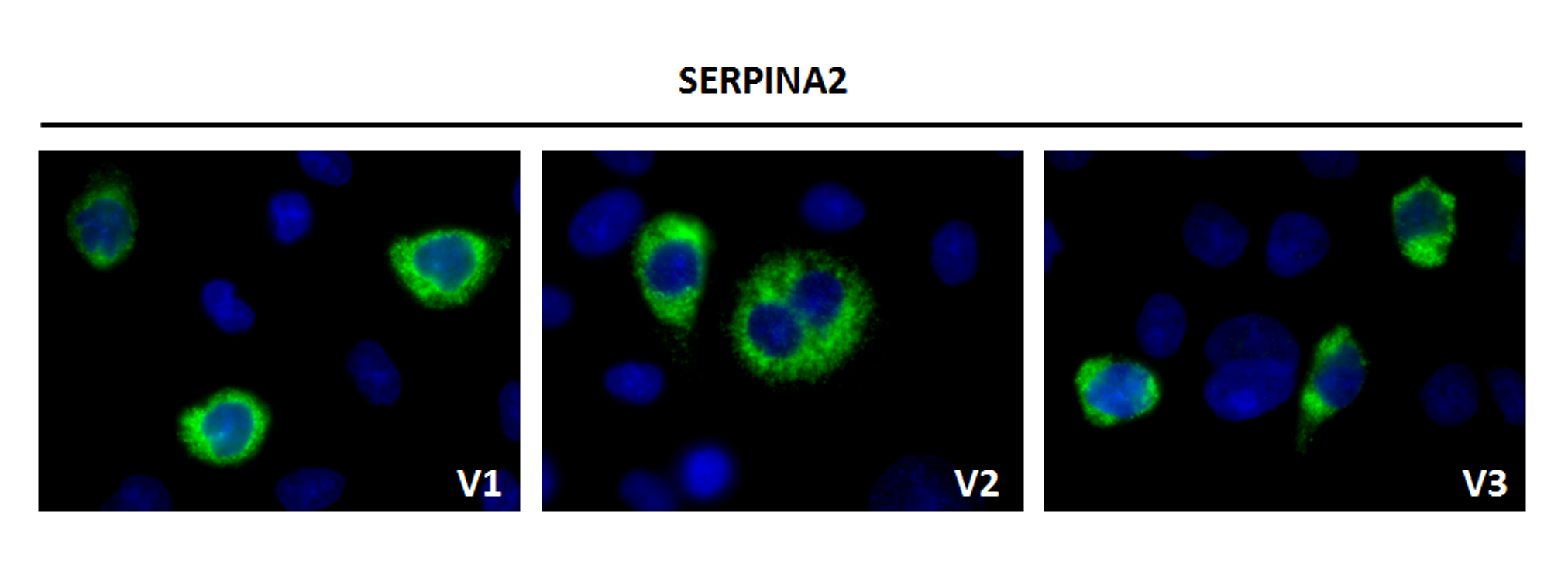

Supplement: Figure S4 — SERPINA2 expression in CHO cells. CHO cells (ATCC number CCL-61) were stably transduced with SERPINA2 (V1, V2 and V3) vectors. SERPINA2 was stained with anti-V5 and Alexa Fluor 488 (green) antibodies. Nuclei were stained with DAPI. Magnification 1000×. (TIF) [file pone.0066889.s004.tif]

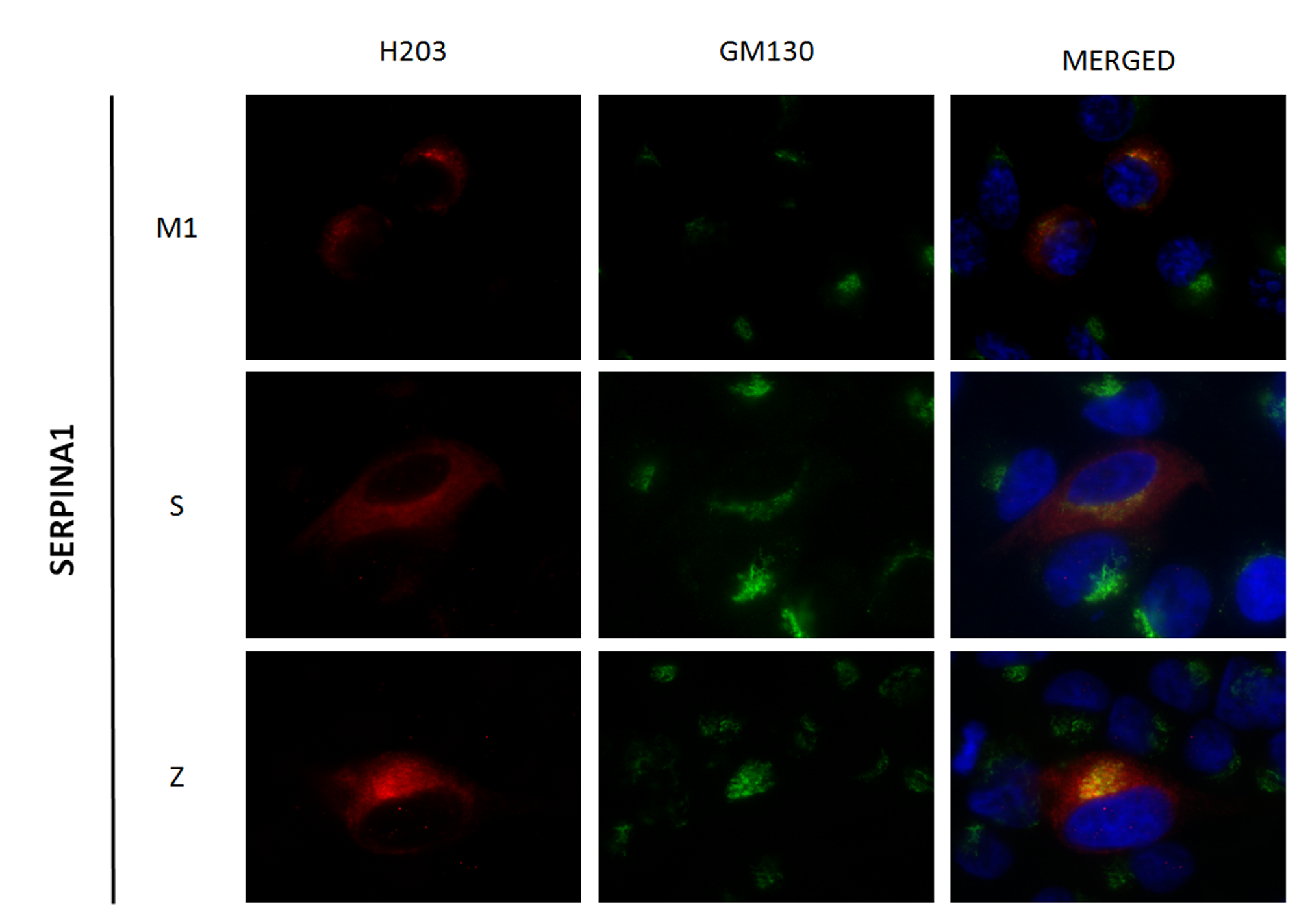

Supplement: Figure S5 — Co-localization of SERPINA1 with the Golgi apparatus. HeLa cells were stably transduced with SERPINA1 (M1, S, and Z) vectors. SERPINA1 was stained with H203 and Alexa Fluor 594 (red) antibodies. Golgi apparatus was detected with GM130 (BD Biosciences) and antibodies Alexa Fluor 488 (green) antibodies. Nuclei were stained with DAPI. Magnification 630×. (TIF) [file pone.0066889.s005.tif]

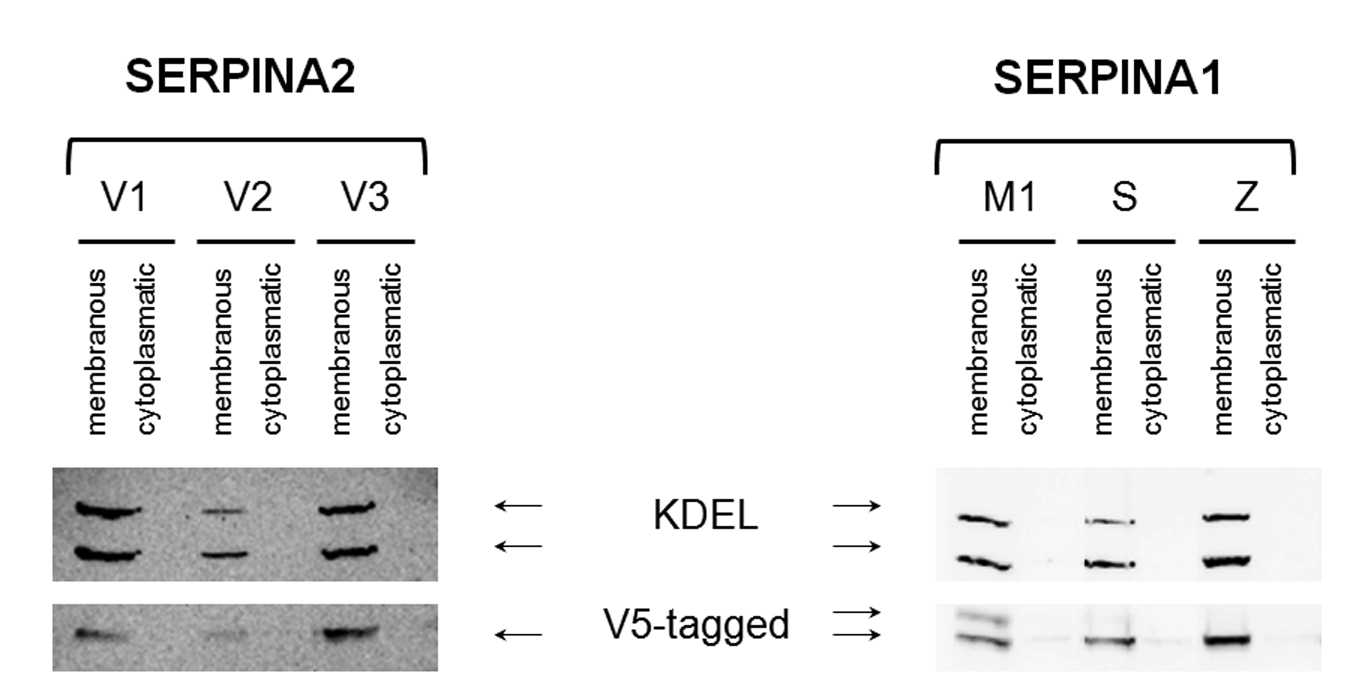

Supplement: Figure S6 — Subcellular protein fractionation of SERPINA2 and SERPINA1. HeLa cells were stably transduced with SERPINA2 (V1, V2 and V3) and SERPINA1 (M1, S and Z) vectors. Membranous and cytoplasmatic fractions were separated by 10% SDS-PAGE. ER protein (KDEL) was detected with anti-KDEL antibody. SERPINA2 and A1 proteins were detected with anti-V5 antibody. (TIF) [file pone.0066889.s006.tif]

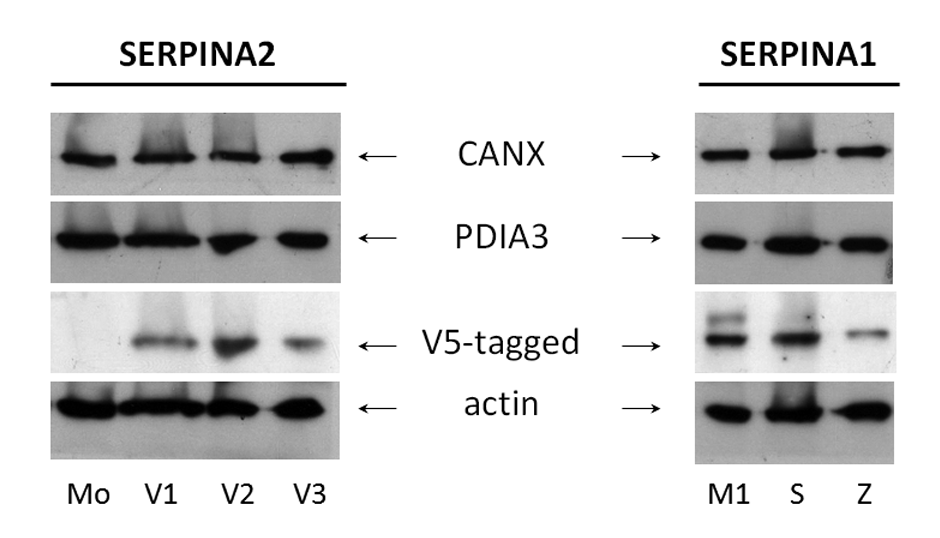

Supplement: Figure S7 — Effect of SERPINA2 and SERPINA1 expression in ER chaperons. HeLa cells were stably transduced with the empty vector (Mo) and with SERPINA2 (V1, V2 and V3) and SERPINA1 (M1, S and Z) vectors. Intracellular lysates were separated by 10% SDS-PAGE. ER chaperones were detected with anti-CANX or anti-PDIA3 antibodies. The other proteins were detected with anti-V5 and anti-actin antibodies. (TIF) [file pone.0066889.s007.tif]

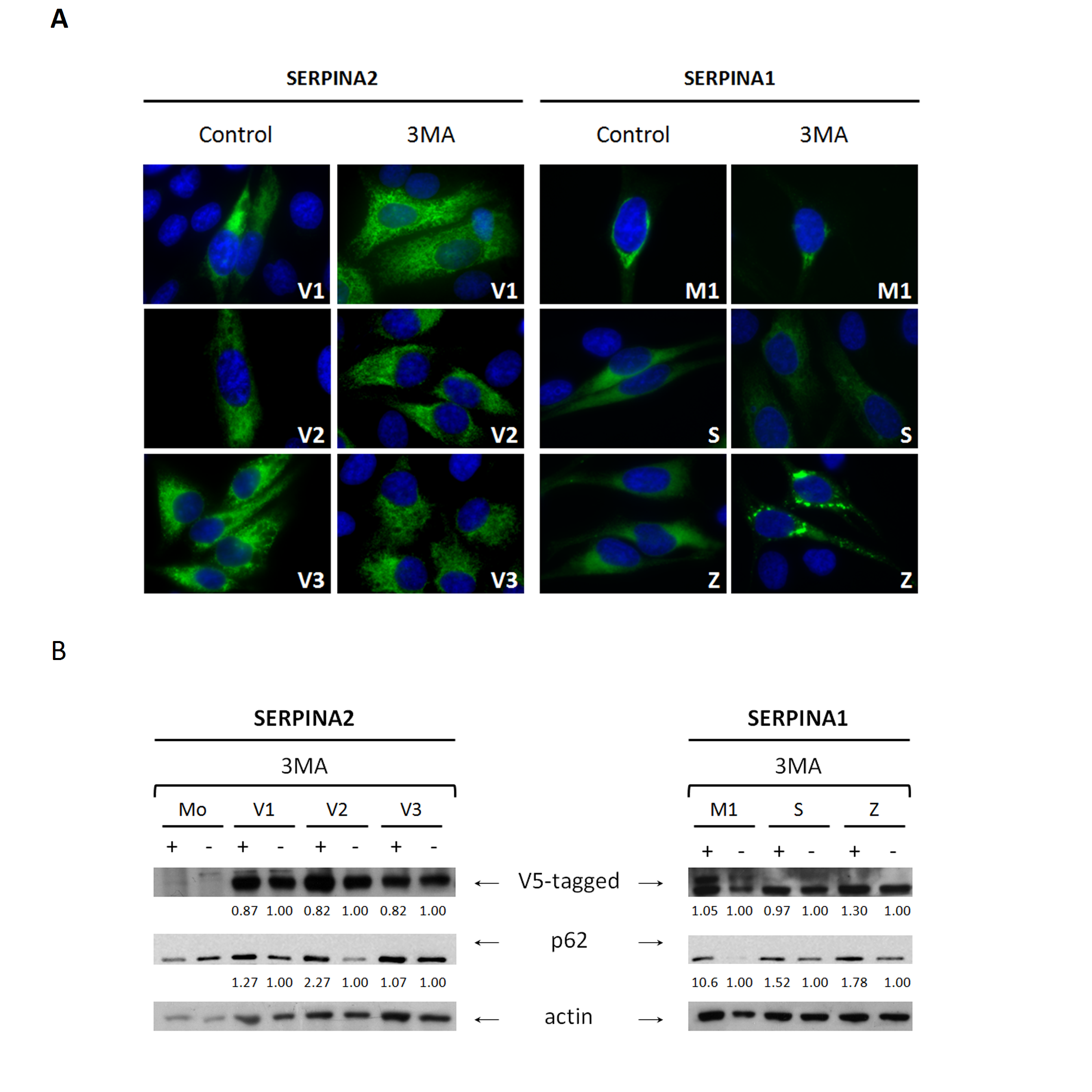

Supplement: Figure S8 — Impact of autophagy inhibition in the expression SERPINA2 and SERPINA1. A - HeLa cells were treated with 10 µM of 3-MA for 24 hours. SERPINA2 and A1 were stained with V5 and Alexa Fluor 488 (green) antibodies. Nuclei were stained with DAPI. Magnification 1000×. B - Intracellular lysates were separated by 10% SDS-PAGE. Proteins were detected with anti-V5, anti-p62 (Santa Cruz Biothecnology) and anti-actin antibodies. The accumulation of the autophagic substrate p62 confirms the inhibition of autophagic response in 3-MA treated cells. The intensity of the bands was normalized against actin and the non-treated sample. (TIF) [file pone.0066889.s008.tif]

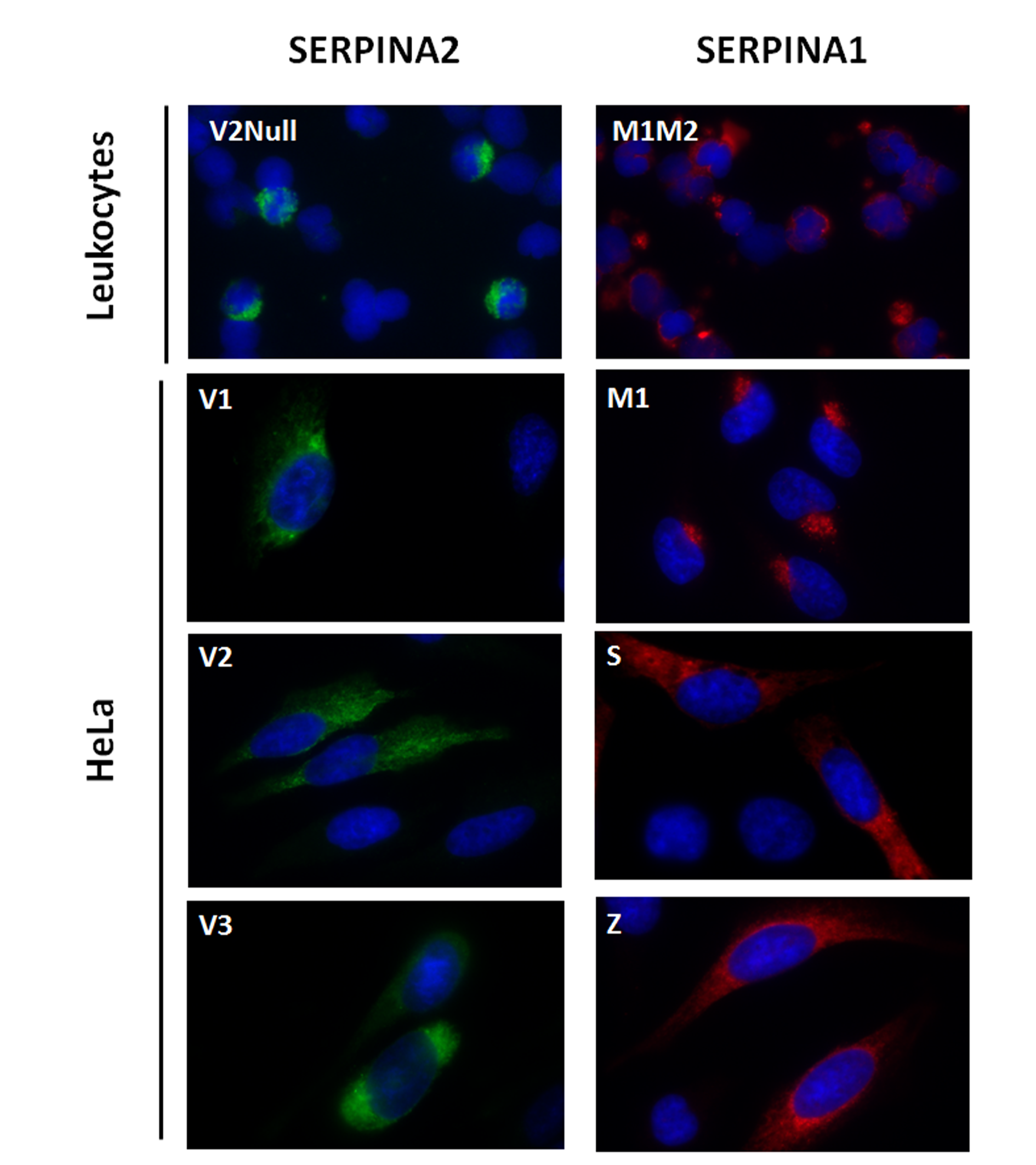

Supplement: Figure S9 — SERPINA2 and SERPINA1 expression in leukocytes and HeLa cells. Leukocytes collected from blood of an individual with a V2Null genotype for SERPINA2 and a M1M2 genotype for SERPINA1. HeLa cells were stably transduced with SERPINA2 (V1, V2 and V3) and SERPINA1 (M1, S and Z) vectors. SERPINA2 was stained with K12 and Alexa Fluor 488 (green) antibodies. SERPINA1 was stained with H203 and and Alexa Fluor 594 (red) antibodies. Nuclei were stained with DAPI. Magnification 630×. (TIF) [file pone.0066889.s009.tif]
