# Supplementary material for: SERPINA2 Is a Novel Gene with a Divergent Function from SERPINA1
Source: PLoS One. 2013 Jun 24;8(6):e66889. doi: 10.1371/journal.pone.0066889 (PMC3691238; doi:10.1371/journal.pone.0066889)
Supplement: Table S1 — Accession numbers of sequences used in the phylogenetic analysis. (DOCX) [file pone.0066889.s010.docx]

**Table S1: Accession numbers of sequences used in the phylogenetic analysis.**

| Species | SERPINA1 | SERPINA2 |
| --- | --- | --- |
| *Homo sapiens* | NM_000295.4 | JX680599 |
| *Pan troglodytes* | XM_003314499 | Absent* |
| *Gorilla gorilla* | JX680603 | JX680600 |
| *Pongo abelii* | NM_001131225.1 | JX680601 |
| *Hylobates sp* | JX680604 | JX680602 |
| *Macaca mulata* | XM_001099255.2 | XM_001098235 |
| *Papio sp* | J00321 | gnl\|ti\|1997424645  gnl\|ti\|1972942700  gnl\|ti\|1922665419 gnl\|ti\|1971909267 |
| *Callithrix jacchus* | XR_089214 | gb\|ACFV01146408.1\|  gb\|ACFV01146409.1\| |
| *Oryctolagus cuniculus* | NM_001171081 | NA |
| *Rattus norvegicus* | NM_022519 | NA |
| *Mus musculus* | NM_001252569; NM_009244; NM_009245; NM_009246; NM_009247 | NA |
| *Canis familiaris* | NM_001080109 | NA |
| *Sus scrofa* | NM_214395. | NA |
| *Bos taurus* | NM_173882 | NA |
| *Ovis aries* | NM_001009799 | NA |
| *Monodelphis domestica* | XM_001375865 | NA |
| *Gallus galus* | XM_421343 | NA |
| *Xenopus tropicalis* | XM_002936063 | NA |

* Seixas et al 2007. NA – not applicable
