# Supplementary material for: SERPINA2 Is a Novel Gene with a Divergent Function from SERPINA1
Source: PLoS One. 2013 Jun 24;8(6):e66889. doi: 10.1371/journal.pone.0066889 (PMC3691238; doi:10.1371/journal.pone.0066889)
Supplement: Table S2 — Polyphen predictions and scores based on SERPINA1 structure for residues with higher probabilities of being positively selected during SERPINA2 divergence. (DOCX) [file pone.0066889.s011.docx]

**Table S2: Polyphen predictions and scores based on SERPINA1 structure for residues with higher probabilities of being positively selected during SERPINA2 divergence.**

| Substitution | Polyphen^a^ | Polyphen2^b^ |
| --- | --- | --- |
| R39K | Benign | BenignScore of 0.003 (sensitivity: 0.98; specificity: 0.44 |
| R196D | Benign | BenignScore of 0.001 (sensitivity: 0.99; specificity: 0.15) |
| P197K | Benign | BenignScore of 0.448 (sensitivity: 0.89; specificity: 0.90) |
| C232D | Benign | BenignScore of 0.000 (sensitivity: 1.00; specificity: 0.00) |
| L241A | Damaging  Score difference: 1.693  Hydrophobicity change at buried site  Normed accessibility: 0.10, Hydrophobicity change: 1.19 | Benign  Score of 0.120 (sensitivity: 0.93; specificity: 0.86) |
| M242Q | Damaging  Score difference: 0.714  Cavity creation at buried site  Normed accessibility: 0.07  Volume change: -79 | Benign  Score of 0.212 (sensitivity: 0.92; specificity: 0.88) |
| E257P | Benign | BenignScore of 0.000 (sensitivity: 1.00; specificity: 0.00 |
| T273E | Benign | BenignScore of 0.004 (sensitivity: 0.97; specificity: 0.59) |
| E279F | Benign | BenignScore of 0.000 (sensitivity: 1.00; specificity: 0.00) |
| M358W | Benign | Benign with a score of 0.003 (sensitivity: 0.98; specificity: 0.44) |
| Q377D | Benign | BenignScore of 0.000 (sensitivity: 1.00; specificity: 0.00) |

^a^ Algorithm predictions made on the basis of annotated non-synonymous polymorphic sites and protein structure and function (Ramensky et. al. 2002)

^b^ Algorithm predictions made on the basis of multi-species alignments, non-synonymous polymorphic sites and protein structure and function (Adzhubei et. al. 2010).
